# Promising strategy for developing mRNA-based universal influenza virus vaccine for human population, poultry, and pigs– focus on the bigger picture

**DOI:** 10.3389/fimmu.2022.1025884

**Published:** 2022-10-17

**Authors:** Nino Rcheulishvili, Dimitri Papukashvili, Cong Liu, Yang Ji, Yunjiao He, Peng George Wang

**Affiliations:** Department of Pharmacology, School of Medicine, Southern University of Science and Technology, Shenzhen, China

**Keywords:** influenza virus, avian influenza, universal vaccine, mRNA vaccine, epidemics, pandemics

## Abstract

Since the first outbreak in the 19^th^ century influenza virus has remained emergent owing to the huge pandemic potential. Only the pandemic of 1918 caused more deaths than any war in world history. Although two types of influenza– A (IAV) and B (IBV) cause epidemics annually, influenza A deserves more attention as its nature is much wilier. IAVs have a large animal reservoir and cause the infection manifestation not only in the human population but in poultry and domestic pigs as well. This many-sided characteristic of IAV along with the segmented genome gives rise to the antigenic drift and shift that allows evolving the new strains and new subtypes, respectively. As a result, the immune system of the body is unable to recognize them. Importantly, several highly pathogenic avian IAVs have already caused sporadic human infections with a high fatality rate (~60%). The current review discusses the promising strategy of using a potentially universal IAV mRNA vaccine based on conserved elements for humans, poultry, and pigs. This will better aid in averting the outbreaks in different susceptible species, thus, reduce the adverse impact on agriculture, and economics, and ultimately, prevent deadly pandemics in the human population.

## Introduction

The outbreaks of influenza viruses recur annually and give rise to worldwide health issues and economic losses (1). Influenza has posed a global threat since the 19th century, resulting in hundreds of thousands of deaths and millions of severe cases annually (2). Indeed, as a zoonotic respiratory disease, influenza has great pandemic potential and poses the threat to humans as well as animals (3) which ultimately increases the reassortment risk of genome segments, transmissibility to humans, and facilitates the emergence of new pandemics. There are four types of antigenically distinct influenza viruses (A, B, C, and D) (4) out of which two types are regarded as health threats. Particularly, influenza A and B are the cause of seasonal epidemics while A provokes pandemics. The reason for global pandemics is the biology of influenza virus itself. Their segmented RNA genome enables frequent antigenic changes (5–7). For this reason, the seasonal vaccines are developed annually based on the expected circulating strains (8) of two influenza A viruses (IAVs)– H1N1 and H3N2, and two influenza B viruses (IBVs)– Victoria and Yamagata lineages. Because of the strain mismatches (9), the effectiveness is usually no more than 60% (10, 11). Conventional vaccines against influenza are also available for veterinary use, however, they are based on specific strains, hence, are highly strain/subtype-specific (12). Recently, nucleic acid vaccines draw special attention. Particularly, mRNA vaccines have been developed to be a highly potent, cost-effective, and safe alternative to conventional vaccines against infectious diseases (13–15). The current coronavirus disease 2019 (COVID-19) pandemic has demonstrated that mRNA vaccines have advantages over nucleic acid vaccines and compared to the other types of vaccines: their production is fast and manufacturing is cell-free; the host cell acts as a factory to produce antigen after the vaccination; it does not pose a risk of transportation into the nucleus and genomic integration as after delivery into the cytoplasm the mRNA is directly translated into the desired antigen protein (16). Therefore, considering its promising potential, the development of an mRNA vaccine against influenza undoubtedly makes sense (17, 18). Importantly, IAVs circulate not only in humans but also in a wide range of animals out of which domestic poultry and swine pose a significant risk for the spread of highly pathogenic strains as well as evolving new subtypes. Similar to the human influenza vaccines, there is no universal vaccine that could elicit protection against all the highly pathogenic avian influenza virus (HPAIV) strains in poultry. HPAIV epizootics have already caused financial loss to agriculture worldwide. The vaccination *in ovo* or intramuscularly (i.m.) seems to protect the chicken from HPAIVs but these vaccines are strain-specific (19). Hence, the absence of broad-spectrum vaccines poses a threat of a panzootic (20). Pigs, on the other hand, often become “mixing vessels” for the origination of the new pandemic influenza viruses as they have receptors for both avian and human IAVs (12, 21), thus, their vaccination is of great importance. Furthermore, the most serious impacts of influenza viruses on humans emerge from the IAVs. Most importantly, HPAIVs have already been evidenced to cross the species barrier to infect humans (21). HPAIVs are characterized by high mortality in poultry and in the human population too. The HPAIVs infections have already occurred in alerting number of humans (22–25). E.g., between the years 2003 and 2022, 865 cases of human infection with only H5N1 were reported worldwide. Out of these cases, 456 were fatal. From 2013 to date, 1,568 confirmed human cases of H7N9 have been reported where 616 were fatal (26). According to the world health organization (WHO), the pandemic of HPAIV seems to be impending (21). Being the first line of defense, the right strategy of immunization is the best available prophylactic measure against IAVs (27). Vaccination of poultry (28) and other vulnerable and key animals along with humans with the universal mRNA vaccine will reduce the excretion of IAV in infected animals as well as raise the threshold of viral load that is necessary for its infectivity. Consequently, it will prevent the spillover on humans and allow to avert epidemics and pandemics. Hence, this review addresses the differentiation of IAV and avian influenza virus (AIV), the current situation of vaccine development, emergence of HPAIVs, and proposes a promising strategy of preventing future epidemics/pandemics and epizootics/panzootics via application of universal mRNA vaccine based on conserved elements.

## Influenza viruses– classification, structure, and emergency

**Figure 1:**
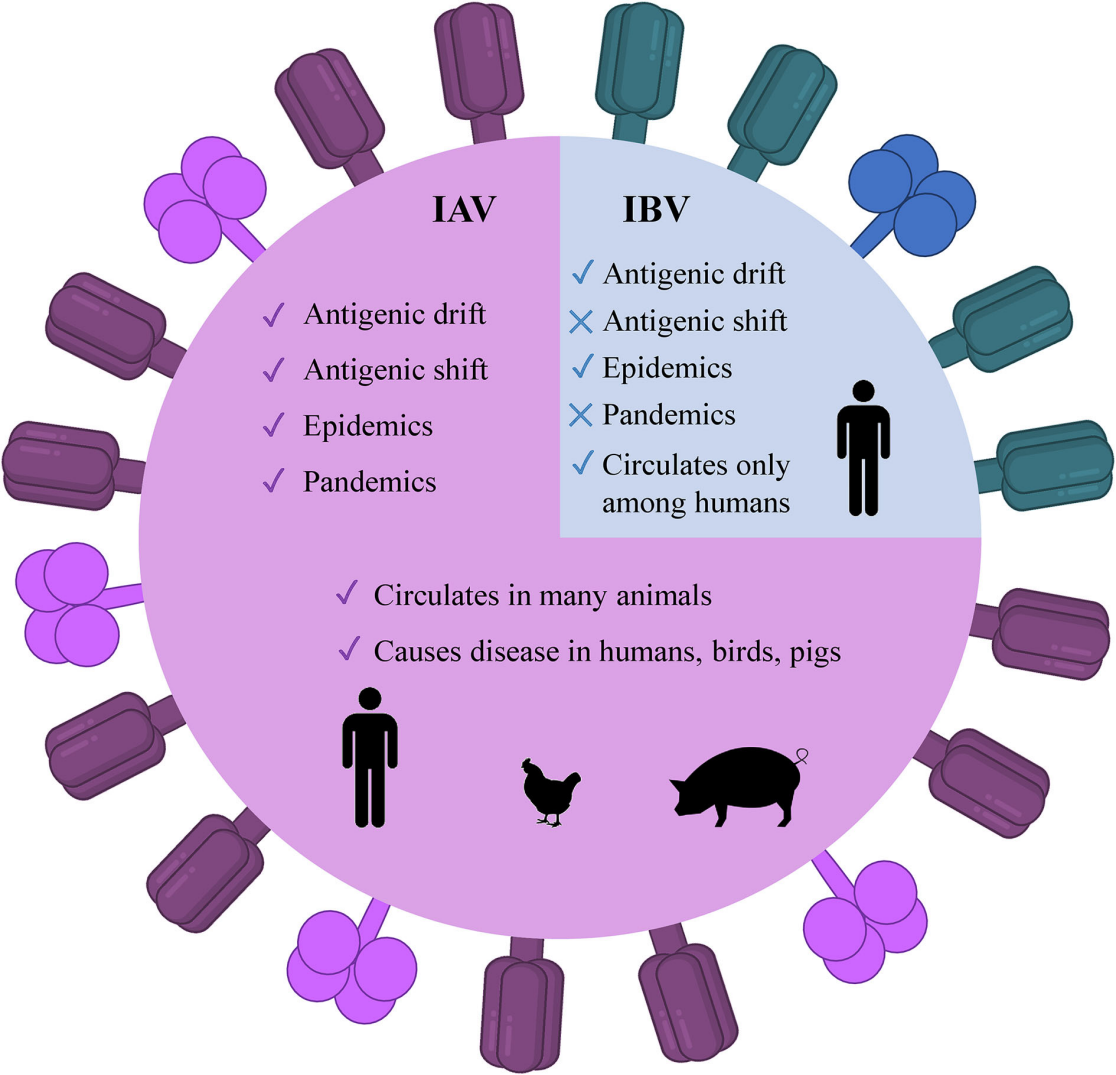
Illustrated schematic differences between IAV and IBV. The trimeric HA (deep purple) and tetrameric NA (magenta) are present as protruded glycoproteins. IAV, influenza A virus; IBV, influenza B virus, HA, hemagglutinin; NA, neuraminidase.

There are four genera of influenza viruses in the family of Orthomyxoviridae– Alphainfluenzavirus, Betainfluenzavirus, Gammainfluenzavirus, and Deltainfluenzavirus. Each of these genera comprises the species– influenza A, B, C, and D viruses, respectively. C and D influenza viruses are not considered to be a threat to humans as type C is not known to cause human epidemics while type D does not infect the human population. On the contrary, IAV and IBV are the types of concern as both of them are the reason for seasonal epidemics that results in up to 500,000 deaths around the world annually (2). Among these two types, IAV is the most prevalent and dangerous that is followed by IBV. The contagiousness of IAV and IBV as well as disease symptoms are similar in both cases. The flu symptoms include fever, cough, sore throat, nasal congestion, fatigue, vomiting, and diarrhea, while the complications are pneumonia, sinus infections, exacerbation of chronic conditions such as asthma, heart failure, etc. (29). The general differences of IAV and IBV are illustrated in **Figure 1**. Influenza viruses are negative-sense segmented RNA-containing viruses. IAV and IBV cannot be distinguished virtually and their genome comprises eight segments of RNA, each is responsible for encoding two different glycoproteins– hemagglutinin (HA) and neuraminidase (NA) that are protruded on the viral surface and play a major role in viral entry and egress, respectively (30, 31); matrix (M2) ion channel protein which enables the proton transport and balances pH across the viral envelope during the entry into the host cell and exit (32); matrix (M1) protein which makes the scaffold beneath the virus membrane and helps the virus in the trafficking of the genome segments in the cell (33, 34); RNA-dependent RNA polymerase complex encompassing one “polymerase acidic” (PA) and two “polymerase basic” (PB1 and PB2) subunits; nucleoprotein (NP) that coats the viral RNA segments (4, 7); nuclear export protein (NEP) which regulates the transport of viral ribonucleoproteins from the nucleus and allows packaging of progeny virions (7). Importantly, the classification of IAVs into subtypes comes from their structure (35). Particularly, it is based on the subtypes of surface proteins HA and NA. There are 18 different subtypes of HA and 11 different subtypes of NA meaning that there are potentially 198 subtypes of IAV out of which 131 have already been detected in nature (35). The classification of influenza viruses along with the influenza viruses of concern is given in **Table 1**.

**Table 1:** Classification of influenza viruses and subtypes of concern.

Genus					
*Alphainfluenzavirus*		*Betainfluenzavirus*		*Gammainfluenzavirus*	*Deltainfluenzavirus*
Species					
*Influenza A virus*		*Influenza B virus*		*Influenza C virus*	*Influenza D virus*
Subtypes		Lineages		Do not cause human epidemics	Usually do not cause illness in human
H1N1 H1N2 H2N2 H2N3 H3N1 H3N2	**H5Nx** H7N1 H7N7 **H7N9** **H9N2**	Victoria	Yamagata		

The highly pathogenic subtypes (HPAIVs) of concern are marked in bold.

## Genetic reassortment, influenza in poultry and pigs

**Figure 2:**
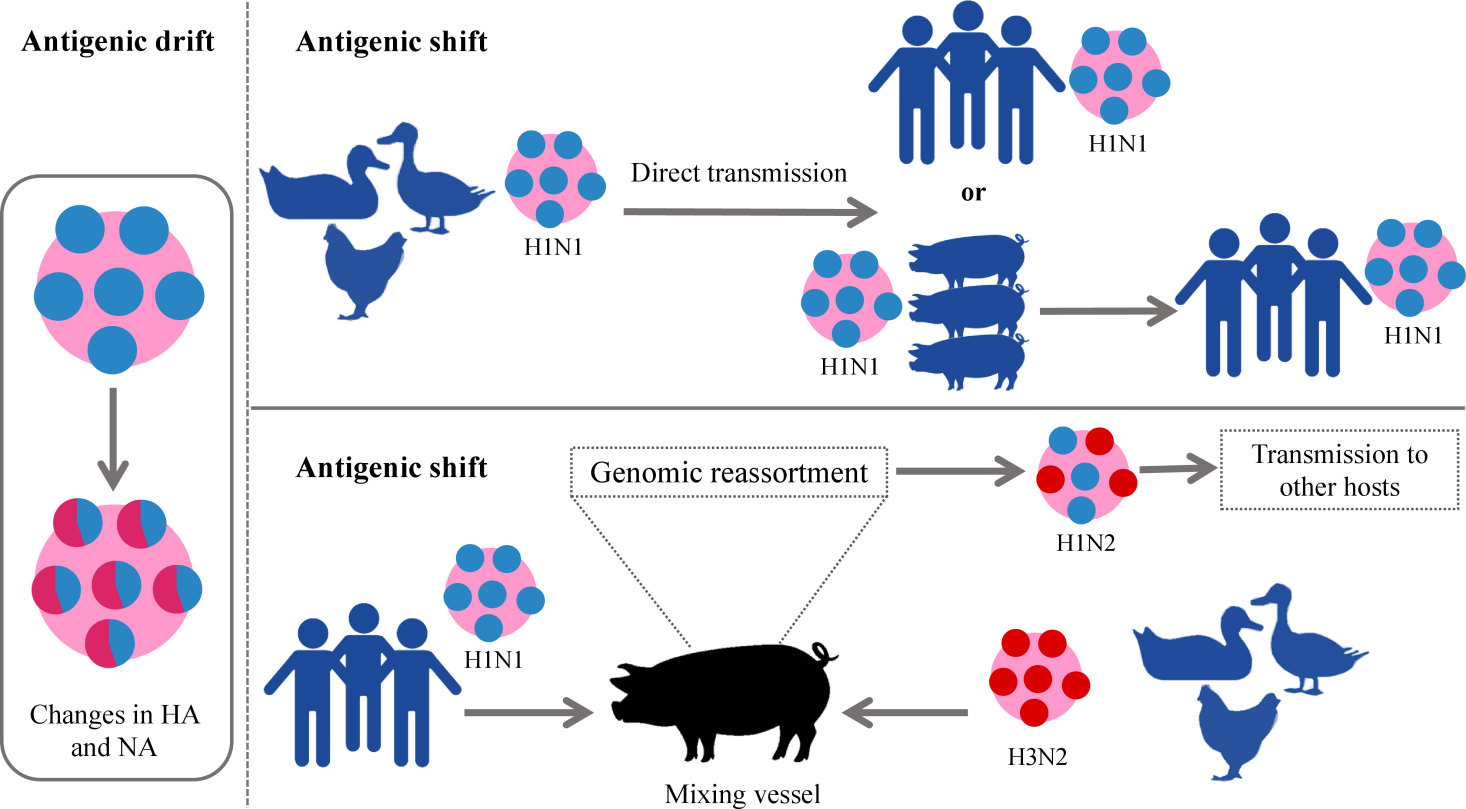
Schematic illustration of antigenic drift and shift in IAVs. In the left panel antigenic drift is demonstrated which results in small changes in viral glycoproteins. In the right panel, two forms of antigenic shift are exemplified. Upper panel: direct jump of IAV strain from an animal to a human. Down panel: More complex form of antigenic shift– two different IAV strains infect the same animal (pig) which becomes a “mixing vessel” and genetic reassortment takes place. The “mixing vessel” then can transmit the new strain of IAV to another species, e.g., humans. IAVs, influenza A viruses.

Among all types of influenza viruses, IAVs are the most prevalent (36, 37), associated with sporadic pandemics, and have the largest animal reservoir (37, 38). Particularly, wild aquatic birds are a large source of IAVs. All known IAV subtypes are found in birds except for H17N10 and H18N11 which have only been detected in bats (39). Usually, the low pathogenic AIV (LPAIV) and HPAIV do not cause any symptoms in wild birds while HPAIV is lethal for domesticated birds (40). Influenza as bird flu was first described in 1978 in northern Italy. It was reported as a highly mortal contagious disease in poultry and was called the “fowl plague”. In the 20^th^ century, it was known that this plague was induced by the virus and later demonstrated to be IAV (39, 40). The extreme divergence of IAVs arises from two major mutational mechanisms that are antigenic drift and antigenic shift. The first implies the accumulation of small mutations that lead to changes in the surface glycoproteins. The latter denotes major abrupt changes caused by a direct jump from an animal strain to humans or the genomic reassortment of RNA segments between two or more influenza strains in the same cell resulting in new HA and/or NA glycoproteins and new unpredictable characteristics (41, 42). Antigenic drift gives rise to seasonal epidemics while antigenic shift emerges the pandemics (43). The names of pandemic influenza strains are generated according to the species where this genomic rearrangement takes place. This is the reason why the pandemic H1N1 was called swine flu in 2009– the pig played the role of mixing vessel for swine, human, and avian viruses (4, 12, 41, 44). On the other hand, except for the important role of the pigs in the adaptation of IAVs to humans and other mammals, their infection itself causes a huge economic loss in pig production worldwide (45). The currently circulating strains H1N1, H3N2, and H1N2 are also called swine flu (45). The schematic illustration of these two processes is given in **Figure 2**.

## Common flu *vs.* avian flu– IAV *vs.* AIV

Kennedy Shortridge– Professor Emeritus at the University of Hong Kong spent his life studying influenza viruses and demonstrated that most of the IAVs, including pandemic H1, H2, and H3 subtypes could be isolated from healthy poultry, meaning that the poultry remained apparently healthy, without manifestation of any clinical signs. Additionally, he postulated that southern China created the optimal environment for interspecies transmission where pigs and ducks lived near humans in the area of rice farms (46). Indeed, H5N1– the most well-known strain of HPAIV was first isolated from a farmed goose in Guangdong Province, China in 1996. This was also corroborated *via* the first human infection of H5N1 in Hong Kong in 1997 with a ~60% mortality rate from the live poultry markets while the poultry itself remained apparently healthy (46). Since then, sporadic spillovers have been reported around the world that account for approximately one thousand infected people with H5 and H7 subtypes since 1997 (47).

To expound on the terms IAV and AIV, it should be mentioned that technically, they are the same. AIV belongs to IAV but more commonly is used to denote the influenza viruses that infect birds and display symptoms. The symptoms and complications of IAV in humans and poultry are listed in **Table 2**. Based on their pathogenicity, AIV strains are divided into two types– LPAIV and HPAIV. Although it has been postulated that the influenza viruses have caused human and animal infections since ancient times (48, 49), the worst pandemic in the world is the so-called Spanish flu which killed 40 to 100 million people worldwide (5, 50, 51) exceeding the death toll during the world war II– the deadliest war in the world. On the other hand, if we consider that H1N1 which is LPAIV was the reason for the mentioned pandemic, we can only imagine what can be the outcome if H5N1 or other HPAIVs spread in the human population massively. Furthermore, the outbreaks in poultry and pigs have caused economic losses. The H5N1 has already given a clear warning of its emergence and to increase pandemic preparedness (21). Initially, H5N1 in chickens caused only mild symptoms, such as reduced egg production and ruffled feathers in poultry, hence, it escaped detection. But after a long time of circulation among poultry, it has become HPAIV with 100% lethality within 48 h after infection (21). Fortunately, when the spillover of H5N1 happened in 1997, killing all the chickens in the markets halted the spread of HPAIV to humans. However, it is just a matter of time before the outbreak takes place again. Indeed, in 2013, another HPAIV H7N9 was transmitted to humans and caused hundreds of deaths since then. Notably, there are other HPAIVs– H5Nx such as H5N6 (52–54), and H5N8 that infect poultry, and inter-species transmission to humans has already occurred (43). Until now, among all the HPAIVs, H5Nx and H7N9 are perceived to pose the highest danger.

**Table 2:** Common flu *vs*. avian flu, symptoms and complications in humans, poultry, and pigs.

	Humans	Poultry	Pigs
**Symptoms**	Fever Cough Sore throat Nasal congestion Fatigue Vomiting/diarrhea	General decrease in activity Reduction of appetite Wet eyes Excessive flock huddling Ruffled feathers Decrease in egg production Coughing	No symptoms Fever Coughing/sneezing Discharge from nose Eye redness
**Complications**	Pneumonia (viral and/or bacterial) Ear infections Sinus infections Exacerbation of chronic conditions	Blueness of the head area Fluid in the comb and wattles Legs bleeding underneath the skin Sudden death	Refusal to eat Breathing difficulties Depression

## Timeline of pandemic IAVs

Pandemics affect the world population in multiple ways. Except for the direct influence on the health and lives of the people, a severe pandemic has a negative impact on all sectors of the economy, including agriculture, manufacturing, rapid price increases, shortages of goods, etc. Hence, pandemics have more-less similar outcomes as the World Wars (55). Evidently, the likelihood of pandemics has been increasing over time along with increased urbanization, global travel, and enhanced exploitation of the natural environment (56). Apparently, this tendency is going on and intensifying over time which also increases the risk of more pandemics. Recent examples are the currently ongoing COVID-19 pandemic caused by severe acute respiratory syndrome coronavirus 2 (SARS-CoV-2) (57), the monkeypox virus outbreak in 2003 which originated from contact with pet prairie dogs (58, 59), and the current multi-country outbreak of the monkeypox (60) when in July 2022 WHO declared it a global health emergency (61).

**Figure 3:**
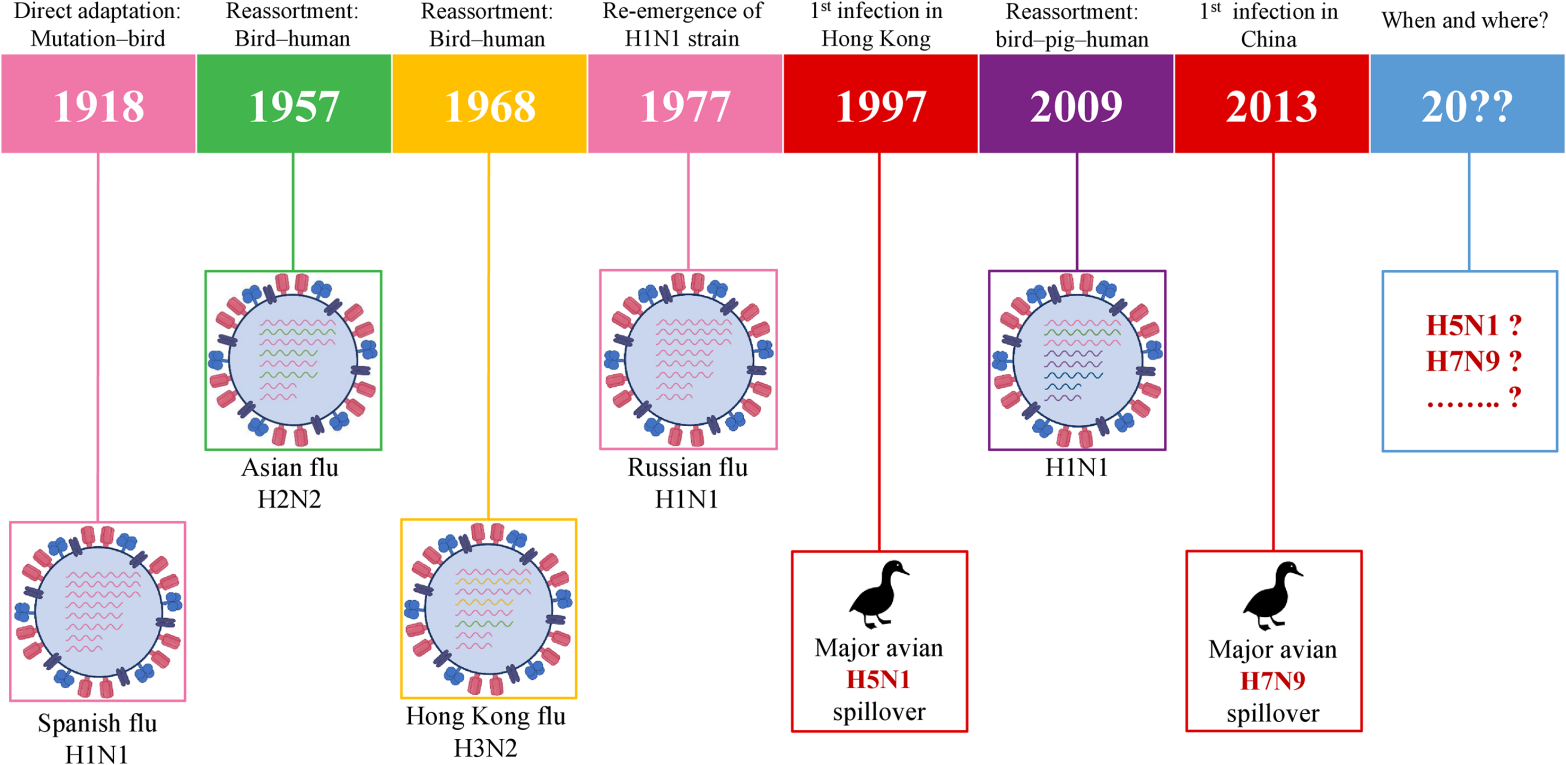
Schematic illustration of pandemic IAVs and IAVs with the pandemic potential. IAV, influenza A virus.

History has been capturing the circulation and emergence of IAVs since the pandemic of H1N1 in 1918, although the actual story of IAVs has begun long before 1918. Following the so-called Spanish flu, the next IAV pandemic emerged in 1957 *via* the spreading of H2N2 in Asia due to the reassortment between avian and human genes of the virus. In 1968, H2N2 was followed by the H3N2 emergence which is called the Hong Kong pandemic (62). In 1977, H1N1 reemerged in Russia (63) which was followed by the swine flu pandemic in 2009. The precursor gene (avian-human-swine) segments have circulated in pigs for over 10 years and, as a result, H1N1/2009 was generated in swine *via* multiple reassortments over time (64). In the meantime, the major spillover events of two HPAIVs– H5N1 and H7N9 took place in 1997 and 2013, respectively. The timeline of IAVs which already caused pandemics and IAVs with pandemic potential is given in **Figure 3**. This chronology demonstrates that it is only a matter of time before a future pandemic will occur. It can be emerged by the accumulation of mutations or genetic reassortment.

## Previous efforts of developing a universal influenza vaccine

The best approach for the prevention of influenza infection is vaccination. Being a global burden due to its high mutation and genetic reassortment capacity, efforts on developing advanced and more universal vaccines for the influenza virus is always ongoing. Since 1938 when the first monovalent IAV vaccine was developed, research on improving the immunization strategy is in the process (65). The current influenza vaccines are quadrivalent and based on two strains of IAV and two strains of IBV as described above. Despite the sufficient safety of the current annually formulated vaccines, they elicit only moderate or low efficacy (65). This is conditioned by the strain mismatches between the circulating strain and the strain which is used for the formulation of the vaccines annually. Currently, the seasonal influenza vaccines are formulated based on the expected IAV and IBV circulating strains for the coming year (8). The current seasonal conventional vaccine technology is egg-based, cell-based, or recombinant. Available conventional vaccines for poultry are based on the following five technologies: The whole AIVs are grown in embryonated chicken eggs which are then chemically inactivated and adjuvanted, HA DNA vaccines, HA protein vaccines or virus-like particles which are produced in insect cells, defective-replicating alphaviruses with H5 gene of AIV, and live virus vectors expressing HA of AIV based on recombinant technologies (e.g., recombinant Newcastle disease virus and Fowl-pox virus) (66). The influenza vaccines for pigs are also based on traditional– inactivated or killed virus-based technology (67, 68). Apparently, all these conventional vaccines are based on specific strains and are highly strain or subtype-specific. Developing the partially or truly universal influenza vaccine that would elicit broad protection remains a big challenge. Currently, there is a number of studies on potentially universal influenza vaccines with promising results in preclinical (69, 70) and clinical development (71–73), however, the universality of the vaccines is limited to subtypes or strains. The ongoing efforts on developing universal vaccines are based on the conserved elements. Indeed, Skarlupka et al., have demonstrated that the influenza vaccine based on the conserved NA elicited protection in BALB/c mice after the challenge (69). Lo et al., have immunized BALB/c mice intranasally with a recombinant adenovirus expressing the conserved NP and M2 antigens of IAV and showed that the vaccine induced a protective and long-lasting immune response (70). Moreover, Nachbagauer et al., performed a randomized, multicenter, placebo-controlled, observer-blind clinical trial to evaluate the immune responses, safety, and immunogenicity of chimeric HA-based vaccine (NCT03300050). The principle of the immunization strategy was to vaccinate the subjects with pre-existing H1 immunity using HA containing H1 head and H1 stalk domains as a prime dose. The second dose of vaccine contained the same stalk but H8 head, while the additional booster dose contained the same HA stalk but H5 head. This way, the chimeric HA-based vaccine induced high anti-HA-stalk IgGs. As the stalk of HA is highly conserved compared to the head domain, it elicited broad reactivity. Vaccination induced a strong, long-lasting, and broad immune response (73). Freyn et al., showed the broad protection induced by the nucleoside-modified mRNA-LNP vaccine based on conserved antigens (HA stalk, NA, NP, and M2) from a viral challenge (10). Evidently, the universality of the influenza vaccines is mainly based on the conservancy of the antigens. However, the protection range of the vaccine candidates developed in previous studies is still limited to specific strains.

## Towards developing the universal influenza vaccine– design of potentially universal influenza vaccine based on conserved elements

The abovementioned information rationalizes the need for a universal vaccine. Therefore, the development of a relatively or truly universal vaccine that will protect the population, the poultry, and swine from the IAV infection is indeed necessary (74). Hence, this type of vaccine could significantly decrease the inter-species transmission risk and advance protection. As IAV is characterized by an extremely high mutation rate, the development of a vaccine that is based on the conserved epitopes of IAV antigens, including the most important strains (H1N1, H2N2, H3N2, H5N1, H7N9) would be rational. On the other hand, considering the recent advancement of nucleic acid vaccines, especially mRNA vaccines (14, 15, 75–77) the formulation of a potentially universal IAV vaccine based on mRNA technology seems reasonable. Indeed, there are mRNA vaccines under development against IAVs (H10N8 and H7N9) that elicit strong humoral immunity in clinical trials (NCT03076385 and NCT03345043) (78, 79).

**Figure 4:**
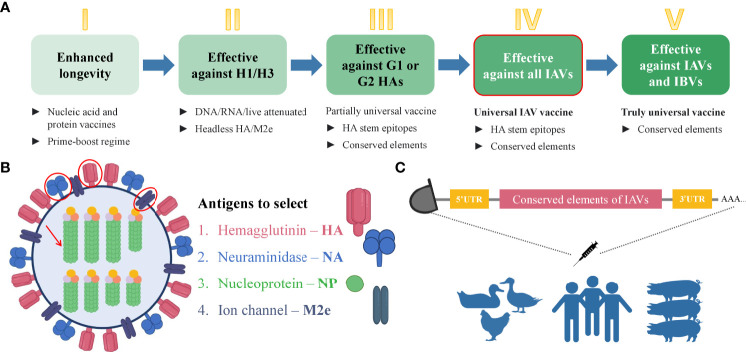
Steps towards developing universal mRNA IAV vaccine. **(A)** Steps that are necessary to gradually achieve the universality of the influenza vaccine. The deeper green color of the box represents the increasing universality of the vaccine. The box with the red outline corresponds to the strategy proposed in this manuscript. Step I– enhanced durability of the influenza vaccine can be achieved by the immunization with nucleic acid and protein vaccines with a prime-boost regime. Step II– a vaccine which will be effective against H1 and H3 subtypes can be based on headless HA and/or M2 ectodomain due to the conservancy of these antigens. Step III– the development of a more universal influenza vaccine that is effective against group 1 (G1) and group 2 (G2) IAVs can be based on conserved elements such as conserved epitopes of selected antigens. Step IV– The development of a universal influenza vaccine effective against all the IAVs can be achieved using the conserved elements. This type of vaccine will be possibly applicable to the human population, poultry, and pigs. Step V – The truly universal vaccine development implies the use of conserved elements with broad protection among all IAVs and IBVs. **(B)** Influenza virus antigens suggested for the selection for developing the universal mRNA IAV vaccine that can be used for the human population, poultry, and pigs. **(C)** Schematic illustration of immunization strategy using mRNA vaccine encoding the conserved elements of IAVs to eliminate the viral shedding and crossing the species barrier. IAV, influenza A virus; IBV, influenza B virus; HA, hemagglutinin; NA, neuraminidase; NP, nucleoprotein; M2e, matrix 2 ion channel ectodomain.

mRNA technology has proved its advantageous properties including favorable safety, low-cost manufacturing, high potency, and rapid development during the current COVID-19 pandemic (14, 15, 75–77). Indeed, the broad application of mRNA-based therapeutics makes an immeasurable advancement for human well-being (17). Since the landmark experiment of Robert Malone and colleagues in 1987 who mixed mRNA with the synthetic cationic lipid incorporated into liposome and observed that the transfection in NIH 3T3 mouse cells and the protein expression was successful (80), it took decades of research until finally, the emergency authorization of mRNA vaccine took place in 2020 for COVID-19 pandemic. During this time, several key achievements were attained that played a crucial role in the development of mRNA-based technology. One of these events was biochemist Katalin Kariko’s work in the 1990s which served the transformation of mRNA into a drug platform. In 1997, she with immunologist Drew Weissman tried to develop an mRNA vaccine against the human immunodeficiency virus (HIV) but resulted in strong inflammatory reactions in mice. Later they developed an excellent strategy for mRNA development *via* altering the part of mRNA– nucleoside modification which allowed mRNA to escape the innate immune response and increase translational capacity (81). The current COVID-19 mRNA vaccines– BNT162b2 and mRNA-1273 developed by BioNTech/Pfizer and Moderna, respectively, incorporate the modified nucleobase N1-methylpseudouridines (Ψs) that enhance immune evasion, protein production, and overall effectiveness (82). Although there was no approved mRNA vaccine until COVID-19 emerged, nowadays, effective mRNA vaccines are used worldwide and unquestionably represent a landmark in vaccine history. The simplicity and time-effectiveness of the mRNA platform among a number of advantages make it a favorable strategy that will solve number of issues related to infectious and non-infectious diseases. For the design of mRNA vaccine, first, the viral antigen(s) should be selected reasonably. The open reading frame (ORF) of the selected antigen will be used in the design of mRNA vaccine. In order to increase the immunogenicity of the vaccine, other immunogen might be adjoined to the mRNA construct. Except for the ORF, the mRNA construct consists of 5’ and 3’ untranslated regions (UTRs), poly-adenosine (poly-A) at the 3’ end which increases the stability of mRNA, and a 5’-cap which provides protection from the degradation and helps ribosomal recruitment (82). Plasmid DNA expressing the gene of interest is synthesized and transformed into DH5α competent *E. coli* strain for amplification. Then it is extracted, purified, and linearized before *in vitro* transcription (IVT) of mRNA is conducted. When protein expression after cell transfection is validated, a delivery system should be applied before the i.m. immunization of experimental animals. The current mRNA COVID-19 vaccines are encapsulated in lipid nanoparticles (LNPs) (13). Ultimately, mRNA is delivered to the target cells where it does not need to be transported into the nucleus, instead, it is directly translated into the cytoplasm, eliminating the risk of genomic integration (16). The body itself becomes a bioreactor of the immunogen that saves time for vaccine production. Moreover, it is degraded by the normal cellular processes and its *in vivo* half-life can be regulated by modifications and delivery methods (14). mRNA can have a certain self-adjuvant effect and induces favorable humoral and cellular immune responses (83). Owing to the capacity to encode any protein of interest, it can recapitulate the expression of the desired influenza antigens similar to the viral infection. Hence, fast, cell-free manufacturing process and production along with safety, efficacy, cost, and time-effectiveness (83) along with other features make mRNA vaccines prominent among nucleic acid and conventional immunization strategies. It is noteworthy that currently there are excellent immunoinformatics tools available to find the conserved sequences in the selected viral antigens (84–86). After the selection of the appropriate conserved amino acid sequences, they can be used to find the epitopes that contain conserved sequences or predict the epitopes on the immune epitope database (IEDB) (87). After the design of the mRNA vaccine which contains the selected conserved epitopes of IAV antigens, the final construct can be optimized by including preferable linkers to adjoin the epitope sequences (88–92), 3’ and 5’ UTRs (93), *in silico* analysis and immune simulation can be performed to predict the immunogenicity, allergenicity, protectiveness, binding capacity to the host receptors, and other necessary features of the vaccine (91, 94–106). *In vivo* study will eventually validate the proposed potentially universal mRNA vaccine against all or selected subtypes of IAVs. Immunization of the human population, poultry, and pigs with the same potentially universal influenza vaccine might elicit protective immunity against various strains of IAVs and eliminate the need for seasonal re-formulation of vaccines. Remarkably, the universal influenza virus vaccine candidates that are based on conserved elements of viral antigens including a stalk of HA (73), NA (69), NP, and M2, are currently in clinical and preclinical development (72). The additional reason and motivation for developing the universal vaccine candidate based on mRNA technology are the successful and effective application of current mRNA vaccines against COVID-19 (75–77). Evidently, effective mRNA vaccines represent a milestone in vaccinology and their application for the development of a potentially universal influenza vaccine candidate based on conserved epitopes seems reasonable. The schematic illustration of the strategy for the development of the universal influenza vaccine is shown in **Figure 4**.

## Summary

Even though much of the work has already been done for the preparedness for influenza outbreaks, the set goals seem to be short-term, and works across many fields lack the focus on the wider picture. The realignment of vaccination strategies as proposed here will work for the common well-being of the human population and animals in terms of pandemic and panzootic prevention. Undoubtedly, it is surpassing to prevent disease in healthy populations than to make an effort to treat disease in an already sick population. Here, we provide the rationale for a potentially universal immunization strategy for overcoming IAV infections in the human population, poultry, and pigs as well as for averting crossing the species barrier. The proposed strategy will aid in advancing the universal vaccine development against IAVs and, at least, will abate the incidence of interspecies spillover events.

## Author contributions

NR and DP contributed to the study conception; NR wrote and prepared the original draft; DP, CL, and YJ reviewed and edited the manuscript; NR and DP visualized the manuscript; YH and PW supervised. All authors contributed to the article and approved the submitted version.

## Funding

This work was supported by the Shenzhen Science and Technology Innovation Program (KQTD20200909113758004).

## Conflict of interest

The authors declare that the research was conducted in the absence of any commercial or financial relationships that could be construed as a potential conflict of interest.

## Publisher’s note

All claims expressed in this article are solely those of the authors and do not necessarily represent those of their affiliated organizations, or those of the publisher, the editors and the reviewers. Any product that may be evaluated in this article, or claim that may be made by its manufacturer, is not guaranteed or endorsed by the publisher.
